# The Human Cytomegalovirus UL116 Glycoprotein Is a Chaperone to Control gH-Based Complexes Levels on Virions

**DOI:** 10.3389/fmicb.2021.630121

**Published:** 2021-04-06

**Authors:** Giacomo Vezzani, Diego Amendola, Dong Yu, Sumana Chandramouli, Elisabetta Frigimelica, Domenico Maione, Marcello Merola

**Affiliations:** ^1^GSK, Siena, Italy; ^2^Department of Pharmacy and Biotechnology (FABIT), University of Bologna, Bologna, Italy; ^3^GSK, Rockville, MD, United States; ^4^Department of Biology, University of Naples Federico II, Naples, Italy

**Keywords:** human cytomegalovirus, gH, UL116, pentamer, gH/gL/gO, chaperone

## Abstract

Human cytomegalovirus (HCMV) relies in large part upon the viral membrane fusion glycoprotein B and two alternative gH/gL complexes, gH/gL/gO (Trimer) and gH/gL/UL128/UL130/UL131A (Pentamer) to enter into cells. The relative amounts of Trimer and Pentamer vary among HCMV strains and contribute to differences in cell tropism. Although the viral ER resident protein UL148 has been shown to interact with gH to facilitate gO incorporation, the mechanisms that favor the assembly and maturation of one complex over another remain poorly understood. HCMV virions also contain an alternative non-disulfide bound heterodimer comprised of gH and UL116 whose function remains unknown. Here, we show that disruption of HCMV gene *UL116* causes infectivity defects of ∼10-fold relative to wild-type virus and leads to reduced expression of both gH/gL complexes in virions. Furthermore, gH that is not covalently bound to other viral glycoproteins, which are readily detected in wild-type HCMV virions, become undetectable in the absence of *UL116* suggesting that the gH/UL116 complex is abundant in virions. We find evidence that UL116 and UL148 interact during infection indicating that the two proteins might cooperate to regulate the abundance of HCMV gH complexes. Altogether, these results are consistent with a role of UL116 as a chaperone for gH during the assembly and maturation of gH complexes in infected cells.

## Introduction

Human cytomegalovirus (HCMV) infects most of the population primarily with an asymptomatic infection in immunocompetent individuals followed by a lifelong latent infection persisting in precursors of dendritic and myeloid cells (Zhuravskaya et al., 1997; Mocarski et al., 2013; Collins-McMillen et al., 2018). Reactivation and re-infection is a serious health problem in immunosuppressed patients where it represents the major causes of severe diseases or fatal outcome (Styczynski, 2018). In transplanted recipients, HCMV accelerates the rate of graft failure and vascular diseases (Streblow et al., 2008). Furthermore, HCMV congenital infection remains a major problem associated to fetus neurodevelopmental delay or hearing/vision defects at birth (Boppana et al., 2013).

This wide plethora of HCMV-associated disease likely relates to the ability of the virus to infect a diverse range of cell types, including epithelial and endothelial cells, fibroblasts, monocyte/macrophages, dendritic cells, hepatocytes, neurons, and leukocytes (Sinzger et al., 2008). This broad cell tropism may reflect the relative abundance of distinct glycoprotein complexes in the virion envelope. Together with glycoprotein B (gB), the gH/gL dimer comprises the “core membrane fusion machinery” conserved among all herpesviruses and is likely to regulate the fusogenic activity of gB (Heldwein and Krummenacher, 2008). However, HCMV encodes a set of proteins that bind alternatively to gH/gL that modify or regulate the activity of the gB-gH/gL core fusion machinery leading to different tropism during the virus spreading in host cells (Zhou et al., 2013). In particular, gH/gL exists on the viral surface as part of a trimeric complex with gO (gH/gL/gO, Trimer) or as a pentameric complex with UL128, UL130 and UL131A (gH/gL/UL128/UL130/UL131A, Pentamer (Huber and Compton, 1997; Ryckman et al., 2008). The presence of the Trimer, although required for entry into all cell types, is sufficient only for fibroblast infection while virus carrying Pentamer greatly expand cell tropism (Zhou et al., 2015) recognizing different cellular receptors. Fibroblast entry relies on Trimer binding to platelet-derived growth factor receptor alpha (PDGFR-α) and ectopic expression of this receptor in PDGFR-α non-expressing cells restores infection of HCMV lacking Pentamer (Kabanova et al., 2016; Wu et al., 2017, 2018). As for this complex, two groups have recently reported the identification of distinct receptors responsible for epithelial tropism. Using a cell-independent screening on purified ectodomain of single transmembrane human receptors, Martinez-Martin et al. (2018) have identified Neuropilin-2, that recognize the pUL128 and pUL131A subunit on the Pentamer, as an essential molecule for HCMV entry. Via a CRISPR/Cas9 genetic screening of human cells, E. et al. identified the 7 TM olfactory receptor OR14I1 associated with G proteins as Pentamer target required for endocytosis of the virus and subsequent infection (Xiaofei et al., 2019).

While differential expression of these receptors influences permissivity to HCMV infection, at least for fibroblasts and epithelial cell types the viral infectivity relies upon the presence of the two gH-based complexes. The relative abundance of Trimer and Pentamer complexes in virions is strain-specific and influence cell tropism and infectivity (Zhang et al., 2018). How the formation of the two gH/gL complexes is regulated at the molecular level remains currently largely unknown, although two recent reports identified two potential players. Li et al. (2015) identified an ER-resident viral protein encoded by the UL148 gene (UL148) that influences the ratio of Trimer to Pentamer and the cellular tropism of HCMV virions. Deletion of UL148 from the viral genome impairs incorporation of the Trimer into virions, leading to a reduced capacity of viral particles to establish infection in fibroblasts, while increasing level of infection in epithelial cells (Li et al., 2015). The opposite outcome was observed by Luganini et al. (2017) using an *US16*-null virus which generated a Pentamer-deprived viral progeny that resulted unable to entry epithelial/endothelial cells. Whether these two viral proteins participate to form a tropism switch during the HCMV life cycle is unknown. However, this finding would imply a more complex system likely involving host proteins.

Among HMCV envelope proteins we identified UL116 as a gH interacting protein that forms non-covalent dimers alternative to gH/gL (Calo et al., 2016). In transient expression, gH and UL116 do not exit the ER unless they are co-expressed. The gH/UL116 complex migrates through the secretory pathway in the absence of other viral subunits suggesting that the antagonism with gL occurs once in the ER. Although the viral envelope localization of UL116 indicates a direct role in viral infection, its competition with any other gH/gL-based complex might reflect a role in the shaping of the envelope complexes.

In this work we found that, in the absence of UL116, cell-free viral spreading is reduced of about 10-folds with evidence of envelope gH/UL116 involvement. We addressed the role of UL116 in the early formation of gH-based viral envelope complexes and its interaction with UL148. We generated HCMV TR strain lacking the expression of either UL116 or UL148 to analyze the contribution of UL116 to complex choice and its potential interaction with UL148. We found that UL116 expression is required for the wt levels of both Trimer and Pentamer in virions produced by fibroblasts and epithelial cells. Furthermore, we also revealed a direct interaction of UL116 with UL148 in cells. These data collectively support the model of UL116 chaperoning gH during the early phases of complexes assembly.

## Materials and Methods

### Protein Purification, Reagents, Plasmids and Antibodies

Trimer, Pentamer and gH/UL116 heterodimer were purified as previously described (Ciferri et al., 2015; Calo et al., 2016).

Primary antibodies: anti-Pentamer was raised by immunizing rabbits with purified whole Pentamer protein (Ciferri et al., 2015) and purifying IgG from the serum over Protein A column. UL116 monoclonal antibodies were produced in mouse following immunization with purified gH/UL116 and hybridomes screening. Two anti-UL116 mAbs were used in this study: clone H4 for immunoprecipitation and clone F11 as probe in immunoblotting. Mouse mAb to Cytomegalovirus IE1 and IE2 (Abcam, ab53495), mouse mAb to Cytomegalovirus pp65 (Abcam, ab6503), mouse mAb to Cytomegalovirus pp28 (Virusys CA004), rabbit pAb to Strep tag (Abcam, ab119810), mouse 6xHis Tag antibody (Invitrogen, MA1-213115), rabbit anti-KDDDDK Tag antibody (Invitrogen, MA1-91878), monoclonal antibody anti-GAPDH produced in mouse (SIGMA, G8795-200UL), mouse Myc Tag monoclonal antibody (Sigma-Aldrich, 05–724), and anti-Strep Tag mouse monoclonal antibody (Invitrogen MA5-17283). Anti-gH human honoclonal antibody MSL-109 was a generous gift of Dr. Adam Feire of the Novartis Institute for Biomedical Research (NIBR, Cambridge, MA, United States).

Secondary antibodies used are: Goat anti-mouse IgG (H + L) highly cross-adsorbed Alexa fluor plus 647 secondary antibody (Sigma-Aldrich, A32728), Goat anti-mouse IgG (H + L) secondary antibody HRP (Invitrogen, 62–6520) and Goat anti-rabbit IgG (H + L) secondary antibody HRP (Invitrogen, 62–6120).

HEK-293T transfections were carried out with Lipofectamine 2000 (Thermo Fischer) according to the manufacturer’s protocol. The HEK-293T transfected cells were trypsinized 48 h post-transfection and treated for immunoprecipitation assays. gHmyc, UL116 and UL148-6XHIS were expressed following cloning of the codon optimized sequence in pcDNA3.1(–) plasmid.

All primers used are listed in Table 1.

**TABLE 1:** Oligonucleotides and synthetic DNA used in this study.

Name	Sequence (5′ to 3′)
UL148_BACnull_Fw	GGGCGGTGGCCGGCACGCCGCATTTCCTAACCCGCGCAGCATGTTGCGCTAACTGTTCACGCTAGGGATAACAGGGTAATCGAT
UL148_BACnull_Rv	CTAGCGTTGACAGACGGCCCGTGGAGGGCCAGTAGGACGAGCGTGAACAGTTAGCGCAACATGCCAGTGTTACAACCAATTAAC
Pre_UL148_Scrn_Fw	CTCCTCATCTTCTGTGGACC
UL148_Nterm_Rv	ATCGGTAGACCAGAAAGGCG
UL148_BACmyc_Fw	GGCTCGCGTTTTCATAACCTACCTGGTGTCGCGGCGTCGGGAACAGAAACTGATTAGCGAAGAAGATCTGTAGGGATAACAGGGTAATCGAT
UL148_BACmyc_Rv	GAACGACGTGTGACGAGGACGTGGTTTCCGCAAGCCTCTACAGATCTTCTTCGCTAATCAGTTTCTGTTCGCCAGTGTTACAACCAATTAAC
UL148_Cterm_Fw	AACGCGCGCCTGACCCGCGG
Post_UL148_Scrn_Rv	GGTTGTAGGTTCGCTACTCG
UL116_BACnull_Fw	ACTTGCCGCTGTACAACGAATTCACCAGCTTTCGCCTGCCCACCTCATGATAGCGGCGGCGTAGGGATAACAGGGTAATCGAT
UL116_BACnull_Rv	AAAGCACAGAGCCAGGAAAAGCAACCAGCCCCGCCATCGCCGCCGCCGCTATCATGAGGTGGCCAGTGTTACAACCAATTAAC
Pre_UL116_Scrn_Fw	CTGTTTGACGCCGTAACCCTGTGC
UL116_Scrn_Rv	AACCGTGGTGGGAGTGGTGACGG
Kan_cassette	ACTTGCCGCTGTACAACGAATTCACCAGCTTTCGCCTGCCCACCTCATGATAGCGGCGGCGTAGGGATAACAGGGTAATCGATTTATTCAACA AAGCCACGTTGTGTCTCAAAATCTCTGATGTTACATTGCACAAGATAAAAATATATCATCATGAACAATAAAACTGTCTGCTTACATAAACAGTAATA CAAGGGGTGTTATGAGCCATATTCAACGGGAAACGTCTTGCTCGAGGCCGCGATTAAATTCCAACATGGATGCTGATTTATATGGGTATAAATGGG CTCGCGATAATGTCGGGCAATCAGGTGCGACAATCTATCGATTGTATGGGAAGCCCGATGCGCCAGAGTTGTTTCTGAAACATGGCAAAGGTAG CGTTGCCAATGATGTTACAGATGAGATGGTCAGACTAAACTGGCTGACGGAATTTATGCCTCTTCCGACCATCAAGCATTTTATCCGTACTCCTGAT GATGCATGGTTACTCACCACTGCGATCCCCGGGAAAACAGCATTCCAGGTATTAGAAGAATATCCTGATTCAGGTGAAAATATTGTTGATGCGCTG GCAGTGTTCCTGCGCCGGTTGCATTCGATTCCTGTTTGTAATTGTCCTTTTAACAGCGATCGCGTATTTCGTCTCGCTCAGGCGCAATCACGAAT GAATAACGGTTTGGTTGATGCGAGTGATTTTGATGACGAGCGTAATGGCTGGCCTGTTGAACAAGTCTGGAAAGAAATGCATAAGCTTTTGCCAT TCTCACCGGATTCAGTCGTCACTCATGGTGATTTCTCACTTGATAACCTTATTTTTGACGAGGGGAAATTAATAGGTTGTATTGATGTTGGACGAGT CGGAATCGCAGACCGATACCAGGATCTTGCCATCCTATGGAACTGCCTCGGTGAGTTTTCTCCTTCATTACAGAAACGGCTTTTTCAAAAATATG GTATTGATAATCCTGATATGAATAAATTGCAGTTTCATTTGATGCTCGATGAGTTTTTCTAATCAGAATTGGTTAATTGGTTGTAACACTGGCCACCT CATGATAGCGGCGGCGGCGATGGCGGGGCTGGTTGCTTTTCCTGGCTCTGTGCTTT
gH_12xHis	ATGAGGCCTGGCCTGCCCAGCTATCTGATCATCCTGGCCGTGTGCCTGTTCAGCCATCTGCTGAGCAGCAGATACGGCGCCGAGGCCGT GTCCGAACCCCTGGATAAGGCCTTCCATCTGCTGCTGAACACCTACGGCAGACCTATCCGGTTCCTGCGCGAGAACACCACCCAGTGCA CCTACAACAGCAGCCTGCGGAACAGCACCGTGGTGCGCGAGAATGCCATCAGCTTCAATTTCTTCCAGAGCTACAACCAGTACTACGTGT TCCACATGCCCCGGTGCCTGTTTGCCGGACCTCTGGCCGAGCAGTTCCTGAACCAGGTGGACCTGACCGAGACACTGGAAAGATACCA GCAGCGGCTGAATACCTACGCCCTGGTGTCCAAGGACCTGGCCAGCTACAGAAGCTTCAGCCAGCAGCTGAAGGCCCAGGACAGCCTG GGCGAGCAGCCTACAACAGTGCCTCCACCCATCGACCTGAGCATCCCTCACGTGTGGATGCCTCCCCAGACCACCCCTCACGGCTGGA CCGAGTCTCACACAACCAGCGGCCTGCACCGGCCCCACTTCAACCAGACCTGCATCCTGTTCGACGGCCACGACCTGCTGTTCAGCACC GTGACCCCATGCCTGCACCAGGGCTTCTACCTGATCGACGAGCTGAGATACGTGAAGATCACCCTGACCGAGGATTTCTTCGTGGTGACAG TGTCCATCGACGACGACACCCCTATGCTGCTGATCTTCGGCCATCTGCCCCGGGTGCTGTTCAAGGCCCCTTACCAGCGGGACAACTTCAT CCTGCGGCAGACCGAGAAGCACGAGCTGCTGGTGCTGGTGAAAAAGGACCAGCTGAACCGGCACAGCTACCTGAAGGACCCCGACTTCC TGGACGCCGCCCTGGACTTCAACTACCTGGATCTGAGCGCCCTGCTGAGAAACAGCTTCCACAGATACGCCGTGGACGTGCTGAAGTCCG GCCGGTGCCAGATGCTGGACAGACGGACCGTGGAAATGGCCTTCGCCTATGCCCTGGCCCTGTTCGCCGCTGCCAGACAGGAAGAGGCT GGCGCTCAGGTGTCAGTGCCCAGAGCCCTGGATAGACAGGCCGCCCTGCTGCAGATCCAGGAATTCATGATCACCTGTCTGAGCCAGACC CCACCCCGGACCACACTGCTGCTGTACCCTACAGCCGTGGATCTGGCCAAGCGCGCCCTGTGGACCCCTAACCAGATCACCGACATCACC AGCCTCGTGCGCCTGGTGTACATCCTGAGCAAGCAGAACCAGCAGCACCTGATCCCTCAGTGGGCTCTGCGGCAGATCGCCGACTTTGCC CTGAAGCTGCACAAGACCCATCTGGCCAGCTTTCTGAGCGCCTTCGCTAGGCAGGAACTGTACCTGATGGGCTCCCTGGTGCACTCCATGC TGGTGCACACCACCGAGCGGCGCGAGATCTTCATCGTGGAAACCGGCCTGTGCAGCCTGGCCGAGCTGAGCCACTTTACCCAGCTGCTGG CCCACCCTCACCACGAGTACCTGAGCGACCTGTACACCCCTTGCAGCAGCAGCGGCAGACGGGACCACAGCCTGGAACGGCTGACCAGAC TGTTCCCTGATGCCACCGTGCCTGCTACAGTGCCTGCCGCCCTGTCCATCCTGTCCACCATGCAGCCTAGCACCCTGGAAACCTTCCCCGA CCTGTTCTGCCTGCCCCTGGGCGAGTCTTTTAGCGCCCTGACCGTGTCCGAGCACGTGTCCTACATCGTGACCAACCAGTACCTGATCAAGG GCATCAGCTACCCCGTGTCCACCACCGTCGTGGGACAGAGCCTGATCATCACCCAGACCGACAGCCAGACCAAGTGCGAGCTGACCCGGA ACATGCACACCACACACAGCATCACCGTGGCCCTGAACATCTCCCTGGAAAATTGCGCCTTCTGCCAGTCTGCCCTGCTGGAATACGACGAT ACCCAGGGCGTGATCAACATCATGTACATGCACGACAGCGACGACGTGCTGTTCGCCCTGGACCCCTACAACGAGGTGGTGGTGTCCAGCC CCAGAACCCACTACCTGATGCTGCTGAAGAACGGCACCGTGCTGGAAGTGACCGACGTGGTGGTGGATGCCACAGATGGCGGAGGCTCTGG CGGCGGAAGTGGCGGAGGATCTCACCACCATCACCATCACGGCGGAGGCAGCCATCATCACCACCACCATTGA
UL148_mycHis	ATGTTGCGCTTGCTGTTCACGCTCGTCCTACTGGCCCTCCACGGGCCGTCTGTCAACGCTAGCCGCGACTATGTGCATGTTCGGCTACTGA GCTACCGAGGCGACCCCCTGGTCTTCAAGCACACTTTTTCGGGTGTGCGTCGACCCTTCACCGAGCTAGGCTGGGCTGTGTGTCGCGACT GGGACAGTATGCATTGCACGCCTTTCTGGTCTACCGATCTGGAGCAGATGACCGACTCGGTGCGACGTTACAGCACGGTGAGCCCCGGCA AGGAAGTGACGCTTCAGCTTCACGGGAACCAAACCGTACAGCCGTCGTTTCTAAGCTTTACGTGCCGCCTGCAGCTAGAACCCGTGGTGGA AAATGTTGGCCTCTACGTGGCCTACGTGGTCAACGACGGTGAACGCCCACAACAGTTTTTTACACCGCAGGTAGACGTGGTACGCTTTGCTC TATATCTAGAAACGCTCTCCCGGATCGTGGAACCGTTAGAATCAGGTCGCCTGGCAGTGGAATTTGATACGCCTGACCTAGCTCTGGCGCCC GATTTAGTAAGCAGCCTCTTCGTGGCCGGACACGGCGAGACCGACTTTTACATGAACTGGACGCTGCGTCGCAGTCAGACCCACTACCTGG AGGAGATGGCCTTACAGGTGGAGATTCTAAAGCCCCGCGGCGTACGTCACCGCGCTATTATCCACCATCCGAAGCTACAGCCGGGCGTTGG CCTGTGGATAGATTTCTGCGTGTACCGCTACAACGCGCGCCTGACCCGCGGCTACGTACGATACACCCTGTCACCGAAAGCGCGCTTGCCC GCAAAAGCAGAGGGTTGGCTGGTGTCACTAGACAGATTCATCGTGCAGTACCTCAACACATTGCTGATTACAATGATGGCGGCGATATGGGCT CGCGTTTTCATAACCTACCTGGTGTCGCGGCGTCGGGAACAAAAACTCATCTCAGAAGAGGATCTGAATATGCATACCGGTCATCATCACCATC ACCATCATCATCACCACCATCACTAG
UL116	ATGAAACGCCGCCGCCGCTGGCGCGGCTGGCTGCTGTTTCCGGCGCTGTGCTTTTGCCTGCTGTGCGAAGCGGTGGAAACCAACGCGACCACCGTGACC AGCACCACCGCGGCGGCGGCGACCACCAACACCACCGTGGCGACCACCGGCACCACCACCACCAGCCCGAACGTGACCAGCACCACCAGCAACACCG TGACCACCCCGACCACCGTGAGCAGCGTGAGCAACCTGACCAGCAGCACCACCAGCATTCCGATTAGCACCAGCACCGTGAGCGGCACCCGCAACACCG GCAACAACAACACCACCACCATTGGCACCAACGCGACCAGCCCGAGCCCGAGCGTGAGCATTCTGACCACCGTGACCCCGGCGGCGACCAGCACCATTA GCGTGGATGGCGTGGTGACCGCGAGCGATTATACCCCGACCTTTGATGATCTGGAAAACATTACCACCACCCGCGCGCCGACCCGCCCGCCGGCGCAGG ATCTGTGCAGCCATAACCTGAGCATTATTCTGTATGAAGAAGAAAGCCAGAGCAGCGTGGATATTGCGGTGGATGAAGAAGAACCGGAACTGGAAGATGATG ATGAATATGATGAACTGTGGTTTCCGCTGTATTTTGAAGCGGAATGCAACCGCAACTATACCCTGCATGTGAACCATAGCTGCGATTATAGCGTGCGCCAGAGC AGCGTGAGCTTTCCGCCGTGGCGCGATATTGATAGCGTGACCTTTGTGCCGCGCAACCTGAGCAACTGCAGCGCGCATGGCCTGGCGGTGATTGTGGCGG GCAACCAGACCTGGTATGTGAACCCGTTTAGCCTGGCGCATCTGCTGGATGCGATTTATAACGTGCTGGGCATTGAAGATCTGAGCGCGAACTTTCGCCGCC AGCTGGCGCCGTATCGCCATACCCTGATTGTGCCGCAGACC

### Binding Assay to HFF-1 and ARPE-19 Cells

For the binding of gH/UL116, Trimer and Pentamer to cells, trypsinized human foreskin fibroblasts (HFF-1) or ARPE-19 were divided in identical aliquot of 3 × 10^5^ cells. Cells were first incubated for 20 min at room temperature (RT) with Live/Dead Aqua diluted 1:400 in PBS, then for 60 min with blocking buffer PBS with 1% Bovine Serum Albumin (BSA) and then with 200 μg/ml of gH/UL116, Trimer or Pentamer recombinant complexes for 60 min at RT. All complexes were 6xHis-tagged. After three washes in PBS, mouse monoclonal anti-His and Alexa Fluor 647-conjugated anti-mouse antibody was used to reveal the binding. A total of 10^5^ cells were analyzed for each histogram using FACS BD Canto II (Becton Dickinson, Heidelberg, Germany).

### Cell Lines

Human foreskin fibroblasts-1 (Human [*Homo sapiens*] skin/foreskin normal fibroblasts; SCRC-1041), MRC-5 (Human [*Homo sapiens*] lung normal fibroblasts; CCL-171), ARPE-19 (Human [*Homo sapiens*] retinal pigmented normal epithelial cells; CRL-2302), HEK293T (Human [*Homo sapiens*] embryonic kidney epithelial cells; CRL-1573) cells were obtained from ATCC. HFF-1 cells were cultured in Dulbecco’s Modified Eagle Medium (DMEM, ATCC 30–2002) supplemented with 15% fetal bovine heat inactivated serum (FBS, ATCC 30–2020), 100 I.U./mL penicillin and 100 mg/mL streptomycin (Penicillin-Streptomycin, internally produced). MRC-5 and HEK293T cells were cultured in Eagle’s Minimum Exential Medium (EMEM, ATCC 30–2003) supplemented with 10% fetal bovine heat inactivated serum, 100 I.U./mL penicillin and 100 mg/mL streptomycin. ARPE-19 cells were cultured in Dulbecco’s Modified Eagle Medium/Nutrient Mixture F-12 (DMEM/F-12, ATCC 30–2006) supplemented with 10% fetal bovine heat inactivated serum, 100 I.U./mL penicillin and 100 mg/mL streptomycin. All cell lines were grown at 37°C with 5% CO_2_.

For western blot analysis, cells were lysed with celLytic (Sigma C2978) in a ratio of 100 μl for 10^6^ cells supplemented with EDTA-free protease inhibitor cocktail (Merck 11873580001).

For kifunensine treatment, six T75 flasks were seeded with HFF-1 cells and infected with HCMV TRG (2 flask) and TRG-*UL116*-null (2 flasks) at MOI of 1. After 24 h, kifunensine (Sigma-Aldrich, K1140) was added to the final concentration of 5 μM in the culture media of three flasks (uninfected HFF-1, TRG infected HFF-1, and TRG-*UL116*null infected HFF-1). The same amount of sterile distilled water was added to the remaining 3 flasks. 48 h after drug treatment, cells were harvested, lysed and treated for western blot analysis under reducing and non-reducing conditions.

### Viruses

A bacterial artificial chromosome (BAC) containing the genome of the HCMV TR strain was obtained from Oregon Health Science University (Murphy et al., 2003) and was integrated with a GFP immediate early expressing gene cassette in the intergenic region between US32 e US33A genes. TR, a clinical HCMV strain derived from an ocular vitreous fluid sample from a patient with HIV disease (Smith et al., 1998), was cloned into a BAC after limited passage in fibroblasts (Murphy et al., 2003). HCMV strain TR-GFP (TRG) and each recombinant virus were propagated in HFF-1 fibroblasts grown to 70–80% confluency, as previously described (Cell Lines, STAR Methods), using infectious supernatants at a MOI of 1. Infection of ARPE-19 cells was performed at a MOI of 5. Infection was visualazed at 24 hpi (hours post-infection) by GFP-fluorescence inside cells. At 100% CPE (or GFP signal) or 50% of cells detached from the plate, medium supernatant was collected and cleared of cell debris by centrifugation for 15 min at 4,000 × *g* at 4°C before aliquoting and storing at -80°C.

To titrate viruses, we used a Titration Assay previously described (Britt, 2010) with minor modifications. In brief, 5-fold serial dilutions of samples were performed in DMEM supplemented with 1% fetal bovine heat inactivated serum and 1 mM sodium pyruvate, and 150 μl of each dilution was applied to duplicate wells of a 96-well flat bottom cluster plate containing 2 × 10^4^ HFF-1 fibroblasts, incubated over-night (O/N) at 37°C with 5% CO_2_ before infection. At 24 hpi, the infected cells were trypsinized and transferred in a 96-well round bottom cluster plate. To evaluate the number of cells with GFP-signal, we performed FACS analysis with BD LRSII Special Order System (Becton Dickinson, San Jose, CA, United States) equipped with High Throughput Sampler (HTS) option. Titer was calculated using the following equation: Titer (IU/ml) = (N × P)/(V × D) [Note: N = Cell Number in each well used for infection day; P = percentage of GFP positive cells (considering the dilution virus exhibiting GFP signal < 40%); V = virus volume used for infection in each well (ml); D = dilution fold; and IU = infectious unit].

### BAC Mutagenesis

To generate recombinant viruses a Two-step Red-mediated recombination method has been used as previously described (Tischer et al., 2006) with minor modifications. BAC TR-GFP was used as starting template. In brief, kanamycin resistance cassette, flanked by I-SceI restriction enzyme cleavage sites, was amplified from pEPkan-S shuttle vector using primers containing homologous regions for the integration in the region of interest. Recombination events were performed with *Escherichia coli* GS1783 strain containing a BAC clone of the HCMV TRG strain, the lambda Red system under the control of a heat-inducible promoter and the I-SceI genes under the control of an arabinose-inducible promoter (Tischer et al., 2010). The first recombination step consists in the electroporation of the purified PCR-amplified cassette in competent, heat-induced GS1783 cells. Positive clones for cassette integration were selected based on kanamycin resistance and screened both by PCR and sequencing. The second recombination was triggered through both heat-shock and arabinose and results in the excision of the kanamycin resistance, leaving the mutation in frame with the gene of interest. Putative clones were screened by PCR, sequenced and analyzed with Vector NTI.

### Reconstitution of Infectious Viruses

To reconstitute the virus MRC-5 fibroblasts were electroporated (nucleofected) using a Cell Line Nucleofector Kit V Lonza (VCA-1003) according to the manufacturer’s protocol. In brief, for each reaction, 1 × 10^6^ freshly trypsinized MRC-5 fibroblasts were pelleted by centrifugation at 300 × *g* for 5 min, washed two times with PBS and then resuspended in a solution containing 1,5 μg of BAC and 0,3 μg of pcDNA3.1-pp71 plasmid premixed with 100 μL of Nucleofector solution (82 μL of Nucleofector solution and 18 μL of supplement). Cotransfection of HCMV protein pp71-expressing plasmid markedly increases the efficiency of virus reconstitution from transfection of infectious viral DNA since pp71 acts as a viral transactivator to help initiate lytic infection (Baldick et al., 1997). The cell suspension was then electroportated using a Nucleofector II (program D-023) and then plated and cultured in DMEM supplemented with 1% fetal bovine heat inactivated serum. 24 h after electroporation, medium was changed and cells were cultured by standard methods. When cells exhibited 100% CPE (or GFP signal, observed with a Zeiss Axiovert 200) or 50% of cells were detached from the plate, medium supernatant was collected and cleared of cell debris by centrifugation for 15 min at 4,000 × *g* at 4°C before aliquoting and storing at -80°C. To determine virus titer the “Titration Assay” has been performed as previously described (Viruses, STAR methods).

### HCMV Virions Purification

The supernatant of infected cells was collected 7 days (HFF-1) or 8 days (ARPE-19) after infection and centrifuged for 15 min at 4,000 × *g* at 20°C to clear all cell debris. Cleared supernatant was transferred to polycarbonate ultracentrifuge tubes under lied with 20% sucrose cushion and centrifuged at 30,000 rpm in a Beckman SW32Ti rotor for 50 min. The virus-containing pellet was solubilized in TX-100 lysis buffer (1% Triton X-100 in PBS) and finally equilibrated in SDS-PAGE loading buffer for western blot analysis.

### Immunoprecipitations

Human foreskin fibroblasts-1 cells were infected at MOI of 1 with HCMV TRG, TRG-UL148-myc, or TRG-*UL148*-null. Infection was allowed to proceed for 6 days and then cells were washed in 1×PBS and lysed with Mammalian CelLytic (Sigma-Aldrich) in presence of protease inhibitors. Five hundred micrograms of total protein extracts were incubated overnight at 4°C with 5 μg of Myc Tag Monoclonal Antibody, anti-UL116 Monoclonal Antibody, or anti-gH Human Monoclonal Antibody. Complexes were pulled down using Dynabeads Protein A/G (Sigma-Aldrich, 14321D) according to the manufacturer’s protocol. Recovered beads were washed in lysis buffer and then boiled for 5 min in 2X SDS-PAGE loading buffer with reducing agent. Eluted proteins were separated on SDS-PAGE and immunoblotting performed as described above.

A similar procedure was applied to recover immunocomplexes from transfected HEK293T cells. 3 × 10^5^ HEK293T cells per well were seeded in a 6 wells plate and incubated O/N at 37°C. The following day, cells were transfected with 10 μg of each plasmid. Extracts were then used for immunoprecipitation procedure using 5 μg of each antibody (gH Human Monoclonal Antibody, myc Tag Monoclonal Antibody and UL116 H4).

### Immunoblotting

Proteins were separated by sodium dodecylsulfate-polyacrylamide gel electrophoresis (SDS-PAGE) on 4–12% polyacrylamide pre-cast gels (Bolt 4–12% Bis-Tris Plus Gels) under reducing or non-reducing conditions. Proteins were transferred to nitrocellulose membranes (iBlot 7-Minute Blotting System, Invitrogen), and membranes were blocked with PBS containing 0.1% Tween 20 (ThermoFisher, TA-125-TW) and 10% powdered milk (Sigma-Aldrich, M7409). Antibodies were diluted in PBS containing 0.1% Tween 20 and 1% powdered milk. For detection of primary antibody binding, horseradish peroxidase-conjugated anti-rabbit or anti-mouse IgG antibodies and the Chemiluminescent Peroxidase Substrate (Sigma-Aldrich, 34578) were used, according to the manufacturer’s instructions. The densitometric analysis of signal intensity in Western blotting was performed with ImageLab software.

## Results

### Construction of HCM TR-GFP Strain Mutants and Cell-Free Viral Growth in Human Fibroblast and Epithelial Cells

**FIGURE 1:**
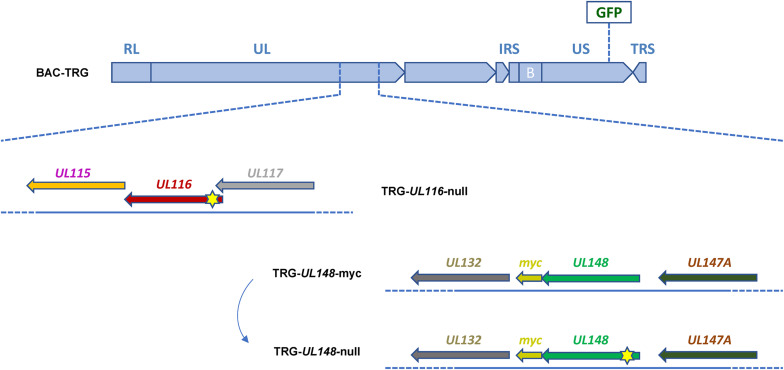
Schematic representation of recombinant HCMV mutants generated. Each mutant is generated through the BAC Mutagenesis Technique. The backbone sequence, common for each mutant shown, consists of the HCMV TR-wt strain cloned into a BAC containing a GFP CDS insertion under control of an Immediate Early CMV promoter, in an intergenic region between US32 and US33A genes (BAC-TRG). The TRG-*UL116-*null clone was generated using the BAC-TRG as template with a single nucleotide insertion in the CDS (between nucleotides in position 4–5) of the *UL116* gene causing a frameshift and a STOP codon formation. The TRG-UL148-myc clone was generated using the BAC-TRG as template inserting the sequence encoding for a myc-tag in frame at Carboxy terminal. The TRG-*UL148*-null clone was generated from the BAC-TRG-UL148-myc by mutation of the codon at position 4 into a STOP codon. Reconstitution of the infectious viruses was performed as detailed in M&M. Yellow stars indicate the approximative position of the stop codon insertion.

To characterize the role of UL116 in viral pathogenesis, we first checked cell-free infectivity in the absence of UL116. We generated a recombinant virus that does not express the UL116 protein by inserting a stop codon close to the *N*-terminus of the UL116 open reading frame (ORF). UL148 is a HCMV ER resident protein reported to bind gH and influence the gH-based complexes formation (Li et al., 2015). We constructed a mutant virus lacking UL148 expression to be studied in parallel. Finally, to detect UL148 in infection, we constructed a recombinant virus expressing UL148-myc tagged protein. In Figure 1 is depicted the map of viruses used in this study. All viruses were generated from the BAC containing the HCMV TR strain into which the GFP gene was introduced between US32 and US33A genes. This template was used to generate recombinant viral genomes via a marker-less two-step RED-GAM BAC mutagenesis (Tischer et al., 2006). The TR-GFP wt (to which we will refer to as TRG) was used to generate the TRG-*UL116-*null, the mutant lacking UL116 expression by insertion of a single nucleotide between residues 4–5 to generate stop codon immediately afterward, and the TRG-UL148-myc, containing the tag at the *C*-terminus. The latter was used as template to generate the TRG-*UL148-*null in which a stop codon was introduced at position 4 of the *UL148* ORF. Each BAC was nucleofected into MRC-5 cells to retrieve the complete mutant virions from cell culture supernatant, according to the procedure detailed in section “Materials and Methods.” Viral stocks were titrated and stored at -80°C for subsequent experiments.

**FIGURE 2:**
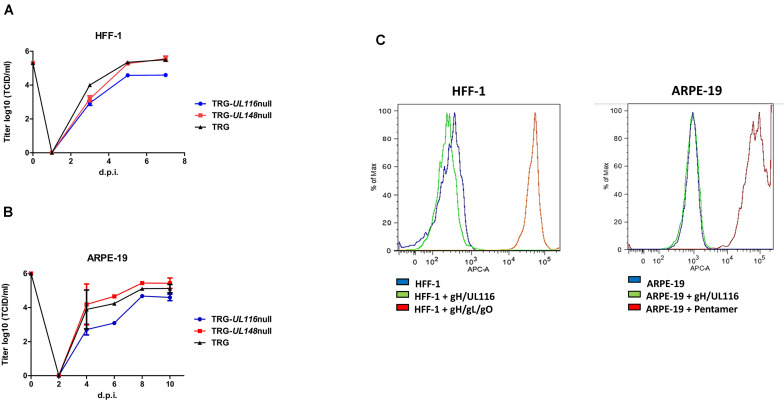
Growth curve of TRG, TRG-UL*116*null, and TRG-UL*148*null and binding of recombinant gH/UL116 to HFF-1 and ARPE-19. **(A)** HFF-1 cells were infected at MOI 1 and cultured for 7 days. At the indicated times, an aliquot of medium were withdrawn and viral titer assessed on fibroblasts. **(B)** ARPE-19 cells were infected at MOI 5 and cultured for 10 days. At the indicated times, an aliquotof the medium were withdrawn and viral titer assessed on fibroblasts. **(C)** 200 μg/ml of recombinant gH/UL116, Trimer and Pentamer were incubated for 1 h with 10^5^ cells in blocking buffer (PBS with 1% BSA). His tag was present on recombinant gH. His-Tag Monoclonal Antibody [HIS.H8] and then Alexa Fluor 647-conjugated anti-mouse secondary antibody were used to reveal complexes bound to cells. A total of 10^5^ cells were analyzed for each histogram using FACS BD Canto II (Becton Dickinson, Heidelberg, Germany).

Fibroblasts have always been the standard cell type for isolation and propagation of HCMV from patient samples and are still the most efficient producer cell line irrespective of the virus strain. At first, we investigated cell-free replication into HFF to verify if the mutations introduced in our recombinant viruses could have an effect on viral growth. A 60–70% confluent 75 cm^2^ flask of HFF-1 cells was infected at a multiplicity of infection (MOI) 1 for each virus. Aliquots of media were collected up to 7 days and used to infect fresh HFF-1 cells in a 96 well plate for viral titration as described (Britt, 2010). 6 log dilution was used for each time point. To assess viral titer, after 24 h of incubation at 37°C, cells were trypsinized and analyzed by FACS to count cells expressing GFP. As shown in Figure 2A, replication of the TR-*UL148-*null virus was identical to the wt while the TRG-*UL116-*null showed a reduction of about 10 times. These data indicate that eradication of UL116 expression influence viral replication and/or infectious ability.

Apart from fibroblasts, epithelial cells are one of the major targets of HCMV infection and are assumed to play an important role during host-to-host transmission since they lay all external body surfaces. We sought to repeat the same analysis on ARPE-19 epithelial cells to verify if mutants had a differential tropism. ARPE-19 cells were infected at a MOI 5 collecting aliquots of media up to 10 days to be used for viral titration as described above. As it can be seen in Figure 2B, viral secretion from epithelial cells displayed 1 day delay in viral secretion compared to fibroblasts but the titers measured at plateau mirrored what was observed in fibroblasts. The TRG-*UL116-*null virus showed 0.65 log lower titer at plateau with respect to the wt or TRG-*UL148-*null. To note that our TR strain not expressing UL148 did not reproduce the behavior reported by Li et al. (2015) who found an increased epithelial tropism.

Results from these experiments suggest that the TR strain impaired cell-free virus production in the absence of UL116 is cell type independent.

### Soluble gH/UL116 Does Not Bind to Fibroblasts and Epithelial Cells

We speculated that virion envelope-bound gH/UL116 dimer might facilitate virus to attach the host cell contributing to viral attachment and/or entry, and therefore lack of UL116 might be responsible for the observed reduction in viral titer of the TRG-*UL116*-null virus. To test this hypothesis, we investigated the binding of the gH/UL116 heterodimer to fibroblasts and epithelial cells. We expressed and purified soluble recombinant gH/UL116 tagged with 6xHis and strep, respectively, Calo et al. (2016) and checked binding to HFF and ARPE-19 cells by FACS analysis. Recombinant Trimer and Pentamer were used as controls. As expected, strong binding of Trimer to fibroblasts and of the Pentamer to epithelial cells were revealed, whereas no binding of gH/UL116 to either cell types could be revealed (Figure 2C). This finding indicates that gH/UL116 does not target a high affinity receptor on cultured fibroblast and epithelial cells.

### Expression Levels of the Major HCMV Envelope Proteins in Infected Cells and Their Incorporation Into Secreted Virions

**FIGURE 3:**
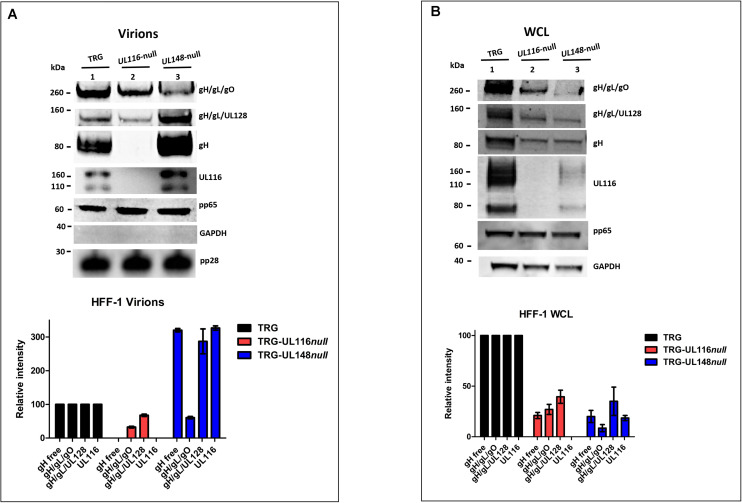
Loss of *UL116* or *UL148* gene products alters the ratio of gH/gL complexes in HFF-1 cellular extracts and virions. UL116, pp65, pp28, and GAPDH were revealed under reducing conditions. gH, gH/gL/gO, and gH/gL/UL128 were probed with anti-Pentamer polyclonal antibodies under non-reducing conditions. Western blots are representative of at least three independent experiments. At the bottom, densitometric analysis of the corresponding immunoblot are shown. Densitometric values of complexes present in the wt virus are considered 100%. The standard deviation indicated in graphs were obtained by the densitometric values from three independent experiments and normalized on the intensity of the pp65 viral marker. Viruses used for infection are indicated on the top of each lane. **(A)** Viral pellets from a T75 flasks were lysed in 50 μl of TX-100 lytic buffer. 5 μl aliquots were run SDS-PAGE under reducing conditions, transfered to a nitrocellulose membrane and probed with anti-pp65. Intensity whose levels of the pp65 bands were used to normalized the consecutive loads. Two aliquots of each sample were then loaded on 4–12% PAGE-SDS under reducing and non-reducing conditions, respectively, and treated for immunoblotting. Virus lysates are indicated on the top of each lane **(B)** Equal amount of total proteins (BCA) from 7 DPI whole cell lysates (WCL) of HFF-1 were separated (NuPage, Invitrogen) and treated for western blot analysis.

Zhang et al. (2018) asserted that the differential tropism and infectivity of distinct strains is also function of the relative levels of Trimer, Pentamer and gH/gL carried by virons. This observation encouraged us to verify the levels of expression of the gH-based complexes in the absence of UL11 or UL148, both in virions and in infected cells. HFF-1 cells were infected with wt and mutant viruses at MOI of 1 for 7 days, then culture media were collected for viral purification on sucrose cushion gradient and cells were harvested. Pelleted virions and cells were then lysed in TX-100 lysis buffer and analyzed by western blot under both reducing and non-reducing conditions. Free gH, Trimer, and Pentamer complexes were separated on SDS-PAGE under non-reducing conditions in which free gH, gH/gL/gO (Trimer), and gH/gL/UL128 (Pentamer) complex migrated at an apparent MWs around 85–90, 260, and 150 kDa, respectively (Figure 3; Ciferri et al., 2015). To reveal free gH and these two complexes, gels were blotted on nitrocellulose membranes and probed with anti-Pentamer that recognizes both gH alone and engaged in disulfide bound complexes.

gH-based complexes immunoblot on extracts from purified virions is shown in Figure 3A. The relative amount of the different species was normalized to the intensity of the capside protein pp28 and of the tegument protein pp65 and provided identical results. Densitometric analysis, graphed on the bottom of the figure, considers values variation of three independent experiments. The TRG wt showed Trimer as major complex on the envelope as previously described (Zhang et al., 2018) and high level of gH although only a minority as gH/gL. As expected, the TRG-*UL148*-null virions showed a strong reduction of virion carried Trimer compared to wt (lanes 1 and 3 of Figure 3A; Li et al., 2015) but also on viral particles derived from the TRG-*UL116*-null mutant an almost comparable reduction was observed (lanes 1 and 2 of Figure 3A). The levels of Pentamer carried by the two mutated viral particles showed opposite outcome. With respect to the wt levels (lane 1 of Figure 3A), TRG-*UL148*-null virions exhibited higher levels of Pentamer (lane 3 of Figure 3A) as previously reported (Li et al., 2015), whereas, in the absence of UL116, considerable reduction of the Pentamer was observed (lane 2 of Figure 3A). Interestingly, in TRG-*UL116*-null mutant virions, the levels of non-disulfide bound gH became undetectable (lane 2 of Figure 3A). This suggests that a relevant amount of the viral gH not engaged in Trimer or Pentamer is normally present on the viral envelope associated to UL116. Finally, the TRG-*UL148*-null virions carried remarkably high levels of gH and UL116, likely as dimer, compared to the wt (lanes 1 and 2 of Figure 3A). Altogether, these data indicate that the absence of UL116 impairs incorporation of both gH/gL complexes in the viral particles whereas the loss of UL148 promotes increased incorporation in virions not only of Pentamer but also of the gH/UL116 dimer.

A representative western blot analysis of infected HFF-1 whole cell lysates (WCL) is displayed in Figure 3B. Densitometric analysis considered values variation of three independent experiments normalized on the intensity of the tegument protein pp65. Both mutants showed a reduced level of Trimer and Pentamer complexes (compare lanes 2 and 3 to lane 1 of Figure 3B). The intracellular pool of free gH in the two mutants were reduced compared to the wt in an almost identical manner and not completely absent from the TRG-*UL116*-null mutant as observed in virions (compare lanes 2 of Figures 3A,B). This suggests that contemporary expression of both UL116 and UL148 is required to completely stabilize intracellular pool of gL-free gH. Viral protein expressed in the cellular extracts of TRG-*UL148*-null mutant infected HFF-1 showed both a pronounced reduction of the Trimer and of UL116 (Figure 3B). These results suggest a close relationship between gH, UL116 and UL148 in the ER of fibroblasts.

**FIGURE 4:**
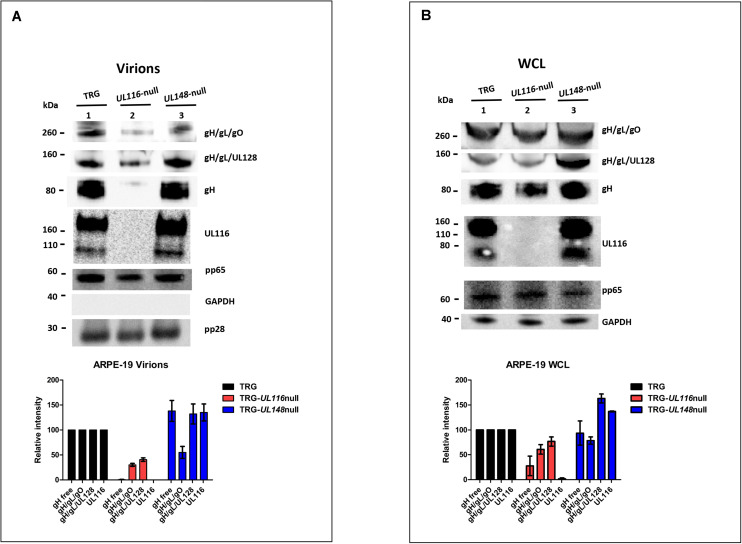
Loss of *UL116* or *UL148* gene products alters the ratio of gH/gL complexes in ARPE-19 cellular extracts and virions. Analysis was performed as specified in the legend of Figure 3. Viruses used for infection are indicated at the top of each lane. **(A)** Immunoblots of lysates from sucrose cushion-purified virions (8 DPI; **B**) Equal amount of total proteins (quantified by BCA) from 10 DPI whole cell lysates (WCL) of ARPE-19 infected with the viruses indicated on the top of the figure were separated on 4–12% PAGE-SDS (NuPage, Invitrogen) and treated for western blot analysis. Graphs reported at the bottom of the figure were generated with values of three independent experiments with statistical variations.

Similar experiments were performed on extracts from infected ARPE-19 cells and virions produced in this cell line. ARPE-19 cells were infected at a MOI of 5 and infection allowed to proceed for 10 days. At the end of this period, cell culture media was used for virus preparations on sucrose cushion while cells were harvested and lysed in detergent containing buffer. Representative western blots from these experiments are shown in Figure 4. Densitometric analysis of the gH-based complexes was mediated on three independent experiments and normalized on the intensity of the pp65 band. The absence of UL116 lead to the disappearance of free gH on virions under non-reducing conditions (lane 2 of Figure 4A) whereas we observed a roughly 50% reduction in infected cells (lane 2 of Figure 4B). These results are consistent with those obtained in fibroblasts (lanes 2 of Figures 3A,B, 4A,B, respectively) and with a role of UL116 in stabilizing and promoting gL-free gH incorporation into virions. The levels of the gH-based complexes carried by TRG-*UL116*-null virions produced by epithelial cells was less than half of both Trimer and Pentamer with respect to the wt (lanes 2 of Figure 4A). As expected, TRG-*UL148*-null virions showed reduced Trimer and increased Pentamer but also higher amount of UL116 (lane 3 of Figure 4A). Thus, unbalanced viral incorporation of gH-based complexes was observed in virion produced after infection of both cell lines and for both mutants.

Analysis of the relative levels of gH-based complexes performed in cell lysates from wt and mutants infected ARPE-19 is shown in Figure 4B. Realtive levels of the viral proteins and complexes were calculated normalizing to the intensity of the pp65 band. TRG-*UL116*-null mutant showed reduced levels of Trimer and Pentamer (lane 2 of Figure 4B) although this reduction was less pronounced with respect to what was observed in fibroblasts (graphs of Figures 3B, 4B). Non-covalently bound gH is present at low levels, the majority likely degraded by the absence of UL116 (Figure 4B, lane 2). The levels of HCMV complexes in ARPE-19 cells infected with the TRG-*UL148*-null mutant differed from what was found in fibroblasts. The levels of Trimer were similar to the wt indicating an intracellular accumulation without productive insertion into virions (Figure 4B, compare lane 1 and lane 3). The intracellular amount of non-covalently bound gH was equal to the wt (lane 3, Figure 4B) suggesting that ARPE-19 may present factors that stabilize this glycoprotein that are absent in fibroblasts.

Taken together, this analysis reveals a similar picture in virion compositions of particles derived from fibroblasts and epithelial cells assigning a crucial role to UL116 and UL148 in generating the pattern of gH complexes typical of the TR HCMV strain. Differences in the intracellular population of HCMV glycoproteins among the two different cell types could suggest a different pattern of interactors that, in epithelial cells, can stabilize HCMV species but not allow their insertion into viral particles. Thus, both proteins may act in increasing proper assembly of gH-based complexes.

### Kifunensine Treatment Partially Restore gH Levels in TRG-UL*116*null Mutant

**FIGURE 5:**
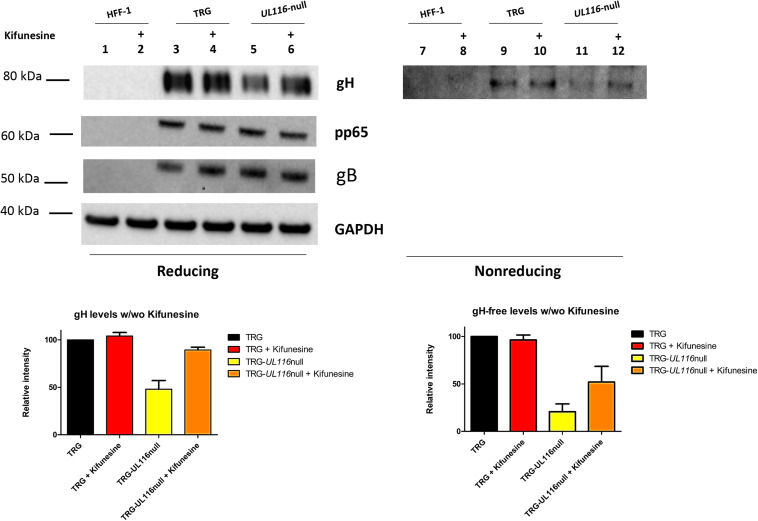
Free-gH levels in WCL from HFF cells infected with TRG and TRG-*UL116*-null under kifunensine treatment. HFF-1 cells were infected in duplicate with TRG wt and TRG-*UL116*-null at an MOI of 1. After 24 h of infection, 5 μM kifunensine was added to one of the pair cultures and incubated for additional 48 h. Cells were then harvested, lysed and WCLs were treated for western blot analysis under both reducing (lanes 1–6) and non-reducing conditions (lanes 7–12). Non infected cells were treated identically and used as control (lanes 1, 2 and 7, 8). Protein separation was achieved on 4–12% NuSieve gels (Invitrogen) using equal amount of total protein (BCA). Samples treated with Kifunensine (lanes 2, 4, 6, 8, 10, and 12) are indicate with a + on the top of the lane. Rabbit polyclonal anti-Pentamer was used to detect gH. Intensity of the GAPDH bands was used to normalize values reported on the graph. Data are reported as mean of five independent experiments with statistical variations.

Data shown so far suggest that UL116 acts as gH “escort” protein implying that in its absence gH would be degraded faster by the endoplasmic reticulum associated degradation (ERAD) machinery. To test this hypothesis, we used the ER mannosidase inhibitor kifunensine that hinders mannose trimming of the oligosaccharide chain and furthers recognition by the ERAD factors (Wang et al., 2011). We reasoned that, in presence of this inhibitor, gH must accumulate in the ER even if not chaperoned. Thus we analyzed extracts from infected cells treated and untreated with this drug. Results are shown in Figure 5. In reducing conditions, the level of gH includes the protein engaged in both difulfide and non-difulfide bound complexes in cellular extracts. Densitometric analysis is shown at the bottom of Figure 5 and was performed on 5 independent experiments. As it can been seen in Figure 5, in TRG wt infected cells, kifunensine treatment did not result in an increase of gH levels under reducing conditions (lanes 3 and 4 of Figure 5). Contrary to this result, in TRG-*UL116*-null infected HFF-1, that presented a gH level of about 48% (average value) compared to the TRG wt (lanes 3 and 5 of Figure 5), inhibition of the ERAD pathway resulted in a gH levels increased to about 89% (average value; Figure 5, lanes 5 and 6). A similar result was obtained under non-reducing conditions in which the kifunensine treatment did not modify the levels of gH in the wt (Figure 5, lanes 9 and 10). In these condition, the base levels of free-gH in the extract derived from TRG-*UL116*-null infection, calculated at about 20% compared to the wt (Figure 5, lanes 9 and 11), raised to 52% upon kifunesine treatment (lanes 11 and 12 of Figure 5). The levels of the two other HCMV proteins, gB and pp65, were not modified by the drug (Figure 5). From these data, we deduced that UL116 increases the amount of intracellular gH through blocking its degradation.

### Co-Immunoprecipitation in Infected and Transfected Cells

**FIGURE 6:**
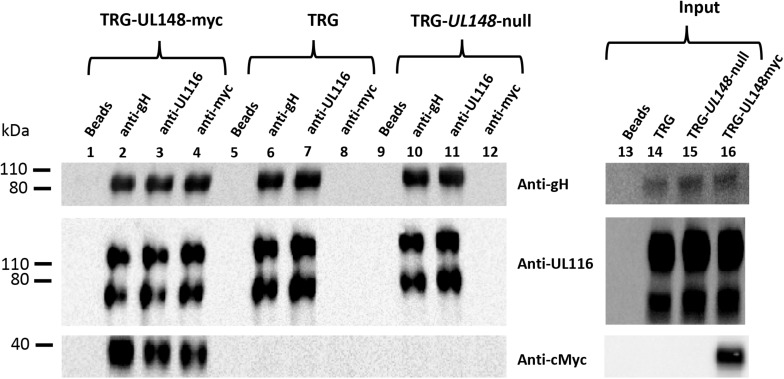
Co-immunoprecipitations of WCL from HFF cells infected with TRG, TRG-UL148myc and TRG-UL*148-*null. HFF-1 cells were infected for 6-days at MOI 1 with TRG, TRG-UL148myc, and TRG-*UL148-*null. Infected and non-infected cell lysates were immunoprecipitated with beads only (lanes 1, 5, and 9), Anti-gH (MSL-109; lanes 2, 6, and 10), anti-UL116 (H4; lanes 3, 7, and 11) and anti-myc to reveal UL148 (lanes 4, 8, and 12) as indicated on the top of the lanes. Protein separation was achieved on 4–12% NuSieve gels (Invitrogen) and probed in western blot by anti-His, anti-myc and mAb F11 for gH, UL148 and UL116, respectively (indicated on the right). GAPDH was used as marker to normalize lysate amount and to exclude contamination in immunoprecipitations. Whole cell lysates (imput, lanes 13–16) were also probed for detection of the individual HCMV proteins.

Our data suggested that UL116 assists gH in its folding indicating a possible interaction with the viral ER resident protein UL148 which was shown to play a role in gH-based complexes choice. We asked if the two proteins could have a direct contact in the early stages of viral glycoproteins assembly. To this aim, we performed co-immunoprecipitation experiments with extracts from HFF-1 cells infected with TRG, TRG-UL148-myc or TRG-*UL148*-null. In the absence of a specific anti-UL148 antibody we used a virus carrying myc-tag at the *C*-terminus of UL148. HFF-1 cells were infected at MOI of 1 with HCMV TRG, TRG-UL148-myc or TRG-*UL148*-null for 6 days. Cells were then collected by trypsinization and lysed in a detergent containing buffer. A small aliquot (1/10 of the total volume) was used to reveal the total amount of the specific proteins (input) while the remaining was split in three identical aliquots and immunoprecipiated with anti-gH (MSL-109), anti-UL116 (H4) or anti-myc (for detection). Immunoprecipitated samples were resolved in SDS PAGE and immunoblotted with specific antibodies to reveal gH, UL116 and UL148. Results are shown in Figure 6. Expression of all individual proteins was revealed by western blot of non-immunoprecipitated WCLs (Figure 6, lanes 14–16). In TRG and TR-*UL148*-null mutant, no UL148 could be detected by the anti-myc antibody and only co-immunoprecipitation of gH by anti-UL116 (Figure 6, lanes 7 and 11) and of UL116 by anti-gH (Figure 6, lanes 6 and 10) could be observed. In extracts infected with the TRG-UL148myc, however, UL148 was co-immunoprecipitated not only by anti-gH but also by anti-UL116 (Figure 6, lanes 2 and 3). Although this result suggests a direct interaction between UL116 and UL148, it does not discriminate whether they interact directly or if they are simultaneously associated to the same protein such as gH that has been reported to bind independently to both proteins (Li et al., 2015; Calo et al., 2016). Our result is consistent with these reports since anti-gH antibodies co-immunoprecipitated both UL116 and UL148 (Figure 6, lanes 2 and 6).

**FIGURE 7:**
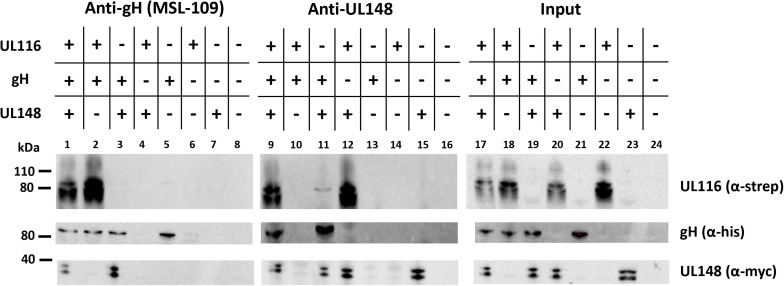
Co-immunoprecipitaions from transfected cells. pCDNA3.1(–)-gH_myc, pCDNA3.1(–)-UL116_strep, and pCDNA3.1(–)-UL148_mycHis were used to transiently transfect HEK-293T cells, either alone or in combination to each other as specified on the top of the figure. pCDNA3.1(–) was used as control (lanes 8, 16, and 24). 48 h after transfection, cells were collected, lysated in TX-100 lysis buffer and the cleared lysates split in two aliquots for immunoprecipitation with anti-gH (human monoclonal MSL-109 antibody) and anti-UL148 (rabbit anti-His). UL116 was revealed by the mouse monoclonal F11 antibody while anti-myc (mouse monoclonal) was used as probe to reveal gH-myc and UL148_mycHis. The western blot shown in figure is representative of three independent experiments.

To provide evidence that UL116 and UL148 have a direct interaction, we performed co-immunoprecipitation in HEK-293T cells co-transfected with expression vectors for tagged individual HCMV proteins. For instance, plasmids used for transient transfection expressed 6xHis tagged gH, His/myc-tagged UL148 and Strep-tagged UL116. 6 wells plates of HEK-293T cells were transfected with different combination of the three expression plasmids (specified on the top of Figure 7). Protein expression was allowed for 48 h before collecting cells and lyse them with lysis buffer. A small aliquot (1/10 of the total volume) was used to reveal protein expression while the remaining was split in two identical aliquots submitted to immunoprecipitations with anti-gH human mAb MSL-109 or rabbit anti-myc for gH and UL148, respectively. Immunocomplexes recovered from magnetic A/G-protein beads were resuspended in SDS-loading buffer and used to load three independent gels. Each nitrocellulose membranes was probed with anti-His, anti-myc and mAb F11 for gH, UL148 and UL116, respectively. Figure 7 shows a representative result of such analysis. Expression of each protein was verified by immunoblotting an aliquot of WCLs (Figure 7, lanes 17–24). As expected, both UL116 and UL148 co-immunoprecipitated with anti-gH (Figure 7, lanes 2 and 3). Co-immunoprecipitation of UL148, via anti-myc antibody, pulled down both gH and UL116 glycoproteins when these species were individually co-expressed with UL148 (Figure 7, lane 11 and 12, respectively).

All together, these results are consistent with a direct interaction between UL116 and UL148, indicating a possible coordination of these two proteins in the ER for the determination of gH-based complexes formation.

## Discussion

In our previous study, we showed that the HCMV UL116 protein is a non-disulfide bonded gH-associated factor alternative to gL and that the complex is inserted into the viral envelope of mature particles (Calo et al., 2016). We sought to further characterize the role of UL116 in the HCMV life cycle by generating UL116-null virus and checking the cell-free infectivity of the progeny. Consistent with the current literature (Dunn et al., 2003; Yu et al., 2003), we found that UL116 is a non-essential protein and that the TRG-*UL116*-null mutant virus was able to infect both fibroblasts and epithelial cells although producing roughly 10- and 6-fold less viral progeny, respectively. The TRG used in this study showed similar cell-free replication in cultured fibroblasts and epithelial cells to the TRG-*UL148*-null mutant. This last result is in contrast with a previous report where the TB40-*UL148*-null virus increases replication in epithelial cells (Li et al., 2015). As it was pointed out in a very recent and elegant report, we suppose that this difference may be due to the genetic background of different strains (Day et al., 2020). The reduction of about 10-times cell-free viral infection in fibroblasts was reminiscent of adhesion factors from other *herpesviridae*. For instance, HSV gC protein acts as a “tethering” factor targeting glycosaminoglycans (GAGs) on cells (Herold et al., 1991) as the very abundant EBV gp350/220 glycoprotein that binds CD35 on host cells (Ogembo et al., 2013). These viral proteins are nonessential for entry but increase about 10-times viral cell-free infectivity. At first, we speculated that the gH/UL116 dimer could recognize a receptor on target cells surface. Therefore, we purified the complex and checked its binding on fibroblasts and epithelial cells by FACS. The data we obtained indicate the absence of a high affinity binding although they could not reveal eventual low affinity binding. Although we cannot exclude that a similar function belongs to gH/UL116, we focused on the intracellular role of UL116.

The TRG wt virus used in this study showed predominant Trimer over Pentamer on virions, consistent with previous reports (Zhou et al., 2015). The relative amount of both complexes was strongly reduced in purified viruses from the recombinant TRG-*UL116*-null mutant, likely not due to a defect in synthesis but rather to an impaired incorporation in infectious virions. We found that gH levels in infected cells are partially rescued following treatment with an ERAD inhibitor indicating that UL116 does act on gH turn over and likely on its correct folding. gH-based complexes in infected cells show a milder reduction, once more suggesting that the impaired step rely on the efficiency of the complexes’ assembly. Indeed, virions derived from the TRG-*UL148*-null mutant were defective in the incorporation of Trimer showing higher levels of Pentamer according to what reported in literature (Li et al., 2015). Differently from ULl48, known to favor Trimer formation versus Pentamer, lack of UL116 impairs both gH/gL derived complexes thus its action must be rather on proper assistance to gH. These findings suggest that UL116 is part of the molecular machinery required for the correct maturation and assembly of the complexes.

The lower amount of Trimer and Pentamer on viral particles reduces but does not abolish cell-free infectivity of the virus, consistent with UL116 categorized as nonessential protein (Dunn et al., 2003; Yu et al., 2003). The group led by Dr. Ryckman has performed a deep analysis on the relationship between the major HCMV envelope glycoproteins and viral infectivity. Among others, their reports showed that the cell-free infectivity is modulated by the relative ratio of Trimer and Pentamer incorporated into the virion and that, although Pentamer definitely control epithelial tropism, its abundance is not straightforward correlated with efficiency of infection in non-fibroblast cell types (Zhou et al., 2013, 2015; Zhang et al., 2018). Trimer alone is sufficient for entry into fibroblasts (Kabanova et al., 2016) whereas Pentamer, always required for infection in all other cell types (Zhou et al., 2015; Kabanova et al., 2016), extends viral tropism through recognition of specific receptors recently identified (Martinez-Martin et al., 2018; Xiaofei et al., 2019). Although cell type restricted receptors explain the tropism specificity, the molecular mechanism responsible for viral infectivity depends on several factors including glycoprotein isoforms, relative ratio of the complexes, RL13 locus and still non identified loci (Stanton et al., 2010; Zhou et al., 2013, 2015; Zhang et al., 2018; Schultz et al., 2020). In our system, we speculate that a 10-times reduction of infectivity, due to the absence of UL116, may be a consequence of the reduced levels of gH-based complexes on viral particles that cannot support priming of the whole population of gB impairing efficient membrane fusion in about 1/10 of the events.

The assembly of gH/gL complexes carried by the mature virions starts during the early phases of glycoproteins assembly in the ER and we addressed the role of UL116 in regulating HCMV glycoproteins assembly. To date, two viral proteins favoring formation of either Trimer than Pentamer have been identified: UL148 and US16, respectively. The single transmembrane (TM) spanning ER resident UL148 protein promotes gO incorporation blocking its degradation by specifically targeting the ERAD receptor Sel1L (Li et al., 2015; Nguyen et al., 2018). This interaction activates unfolding protein response (UPR) leading to an ER expansion whose benefit for viral replication remains unclear (Siddiquey et al., 2018). US16 is a 7TM HCMV protein identified as tropism factor whose absence impairs viral replication in epithelial/endothelial cells at the level of entry or post-entry (Bronzini et al., 2012). Remarkably, US16 is required for incorporation of UL128–131 showing a direct interaction only with UL130 (Luganini et al., 2017). UL148 and US16 favor the incorporation of either gO or ULs, respectively, harmonizing the correct formation of envelope complexes and highlighting that the broad tropism is due to a fine regulation of complexes levels.

**FIGURE 8:**
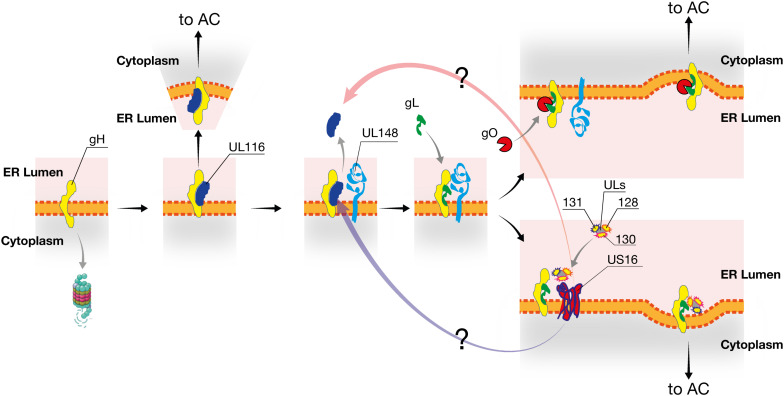
Model of HCMV interaction in the early phases of gH complexes assembly. UL116 is the first interactor of gH in the ER and chaperones the early folding steps. UL148 is recruited through either gH or UL116 and favors the binding of gL and successive association of gO. At limiting availability of UL148, for example engaged by Sel1A, UL116 remains bound to gH and traffic through the secretory pathway reaching the assembly complex and then the mature virion. UL116 can also be released from gH in the absence of UL148, either at low efficiency or by the intervention of US16 or an unknown factor, allowing gL binding and favoring further incorporation of UL128-131 versus gO. Interaction of US16 with UL116 or other HCMV proteins is merely speculative.

From these data, we propose the following model of viral proteins interactions during the early ER post-synthesis phase (Figure 8). We hypothesize that UL116 is the first interactor of gH, stabilizing the protein and protecting it from degradation. To note, gL (*UL115*) and *UL116* are adjacent genes on the same transcription unit and the two proteins are synthesized simultaneously. Next, UL148 interacts potentially with both gH and UL116 mediating binding to gL. This process, in the absence of UL116, occurs at a lower yield. It is possible that the formation of the gH/UL116 dimers rises while UL148 is involved in interaction with other factors such as the ERAD component Sel1L (Nguyen et al., 2018). In the absence of UL148, formation of the Trimer is impaired favoring not only increasing levels of Pentamer (Li et al., 2015) but also promoting stable association of the heterodimer gH/UL116. Two disulfide bonds lock gL to gH and an additional cysteine on gL establishes an alternative disulfide bridge to gO or UL128/UL130/UL131A resulting in the formation of trimeric or pentameric complex, respectively (Ciferri et al., 2015; Chandramouli et al., 2017). Thus, the non-covalent nature of gH/UL116 binding is ideal to chaperon gH toward a native or near-native conformation inducing stable conformer able to avoid host ERAD but also to be conformational competent to bind gL. Conformational instability of gH in the absence of UL116 would explain why the levels of both Trimer and Pentamer were lowered in *UL116*-null virions. UL148 would act downstream of UL116 as a regulatory factor favoring gO incorporation on gH/gL. The role of US16 could be to stabilize the UL128/130/131A making this trimer available for incorporation. Although possible, any interaction between UL148 and US16 remains hypothetical. Intriguingly, the HCMV US17 gene product, known to interfere with the host innate immunoresponse, seems to play a role in controlling the viral level of gH. Gurczynski et al. (2014) showed that the reconstituted AD169 knocked out of the US17 gene show about 3 times reduction of viral gH without impairment of fibroblasts infectivity. Similar to UL148, US17 interferes with the ER stress response inducing aberrant expression of several genes in this pathway. However, no data are available on a direct binding of this protein to gH and the observed reduction of gH levels in the US17 knock-out mutant could be an indirect effect such as an altered trafficking to the assembly complex (Gurczynski et al., 2014).

Similar to UL148 and US16, UL116 is also a nonessential viral protein (Dunn et al., 2003; Yu et al., 2003), highly conserved among strains that may suggest multiple interaction with other host and viral proteins (Foglierini et al., 2019). Our findings demonstrate that UL116 is required for reaching wt levels of both gH-based complexes but more generally of the viral particles’ levels of gH. Remarkably, in the *UL116*-null virus non disulfide bond gH in viral particles was completely missing and its intracellular amount was drastically reduced likely due to accelerate gH degradation. The presence of non-covalently linked gH was firstly revealed by Britt and collaborators while they identified a gp125 glycoprotein, then named gO, as part of gH/gL complex (Li et al., 1997). Additionally, early characterization of the Pentamer by Wang and Shenk, preliminary described as gH/gL/UL128/UL130 complex, revealed a huge amount of non-covalently linked gH in infected cells (Wang and Shenk, 2005). In this work we have shown that this fraction corresponds to gH associated to UL116 and that, in addition, the gH/UL116 dimer represents a consistent fraction of total gH complexes carried by virions at least in TR. The direct interaction between UL148 and UL116 shown here suggests that the two proteins compete for gH association and that the formation of the disulfide bonds with gL is induced by UL148. Indeed, in the absence of UL148, the amount of gH/UL116 dimer further increases on viral particles as well as intracellularly. As UL116 protects gH from degradation in the ER, its meets the definition of molecular chaperone/escort as “any protein that interacts with and aids in the folding or assembly of another protein” and increase the yield of its client (Kim et al., 2013). However, a molecular chaperone is not part of the final complex (Kim et al., 2013) while UL116 heterodimerizes with gH and is found on the viral envelope. Although we have not been able yet to reveal a role for the virion-carried gH/UL116. Our data show that UL116 is a newly identified player of the molecular machinery responsible for the efficient folding and incorporation of gH-based complexes into virions.

## Data Availability Statement

The raw data supporting the conclusions of this article will be made available by the authors, without undue reservation.

## Author Contributions

DY, SC, DM, and MM were involved in the conception and design of the study. GV and DA acquired the data. GV, DA, EF, DM, and MM analyzed and interpreted the results. All authors were involved in drafting the manuscript or revising it critically for important intellectual content. All authors had full access to the data and approved the manuscript before it was submitted by the corresponding author.

## Conflict of Interest

DY, SC, EF, and DM are employees of GSK. DY and DM report ownership of GSK shares and/or restricted GSK shares. GV and DA are or were Ph.D. students sponsored by GSK Vaccines. MM is an employee of the University of Naples Federico II with a consultancy contract with GSK. This study was entirely sponsored by GSK Vaccines. GSK took responsibility for all costs incurred in publishing.
